# The Dreaded Black Line

**DOI:** 10.5334/jbsr.3050

**Published:** 2023-09-07

**Authors:** Mohammed Jasim M. Al-Janabi, Nirav Gupta, Seema Döring

**Affiliations:** 1 UZ Brussel, BE; 2 Lokmanya Tilak Muncipal Medical College, Mumbai, IN

**Keywords:** The dreaded black line, Stress fracture, Fracture, Tibia

## Abstract

**Teaching Point:** “The dreaded black line” is a subtle radiographic sign for a unique type of high-risk stress fracture in anterior tibial cortex.

## Case History

A 19-year-old male athlete was referred for magnetic resonance imaging (MRI) for anterior lower leg pain, aggravated by exercising, relieved by resting, and with history of similar previous episodes.

**Figure 1:**
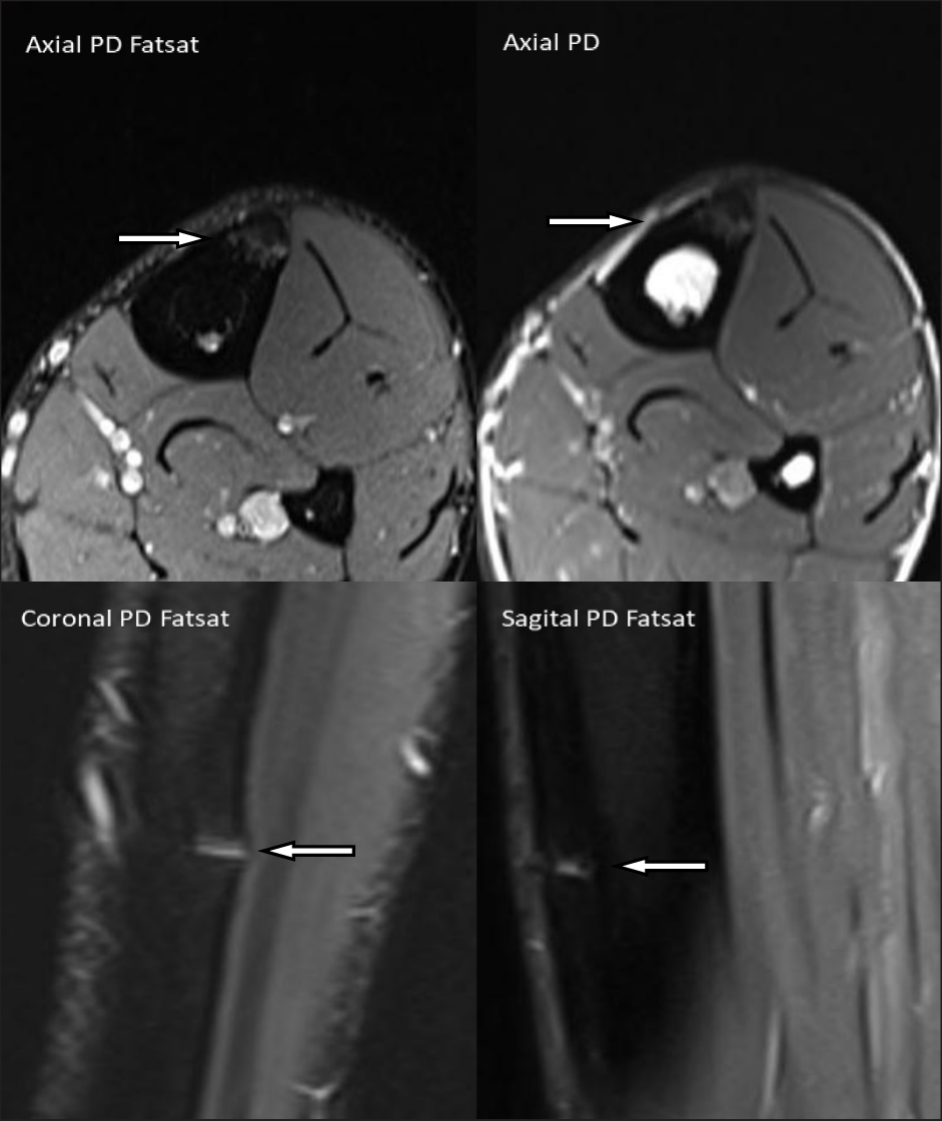


MRI (Figure 1) shows a discrete hyperintense line in the anterior cortex, mid-tibia (arrow). MRI was of limited diagnostic value due to lack of adjacent reactional bone marrow and soft tissue oedema. It is barely visible on axial images due to transverse orientation of the fracture and partial volume effect, and longitudinal images need to be carefully inspected.

**Figure 2:**
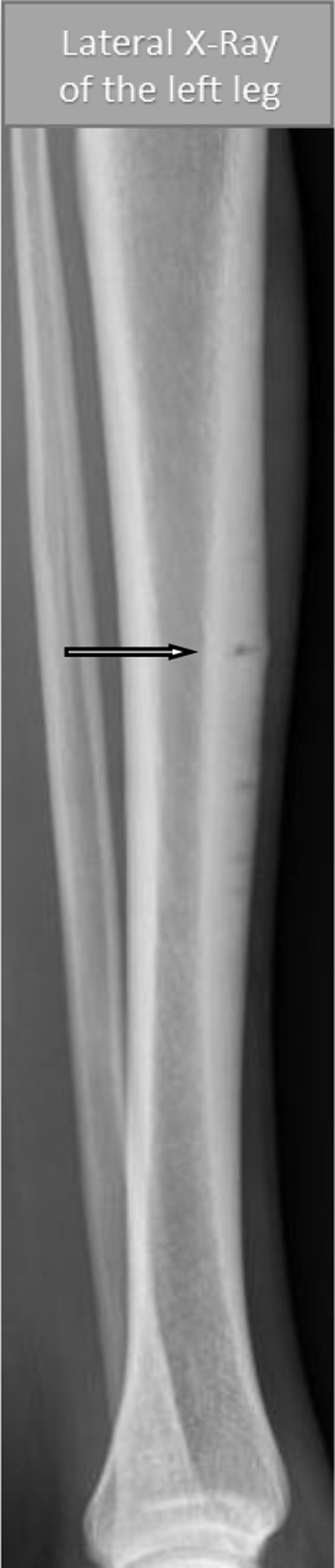


**Figure 3:**
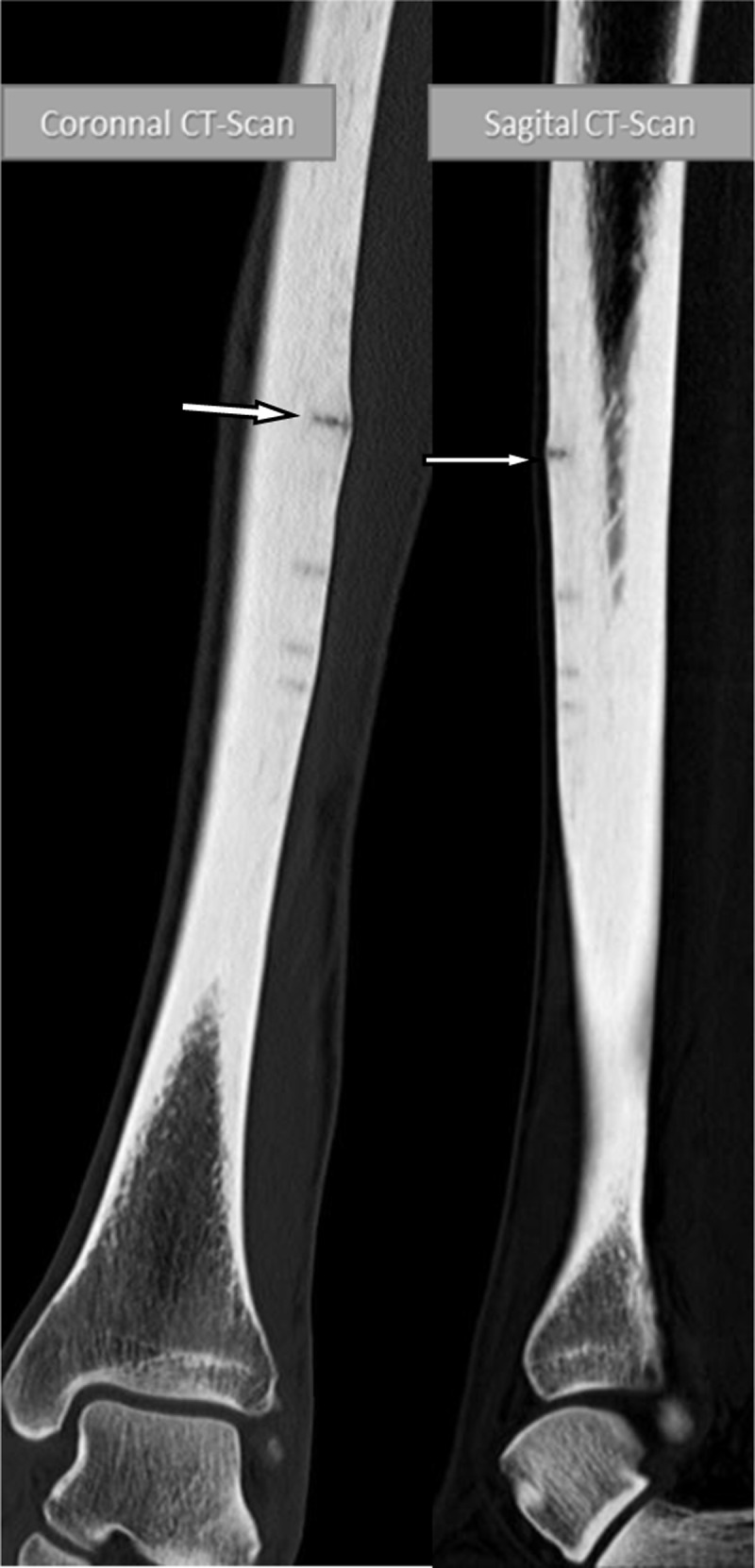


Additional X-ray (Figure 2) and computed tomography (CT) (Figure 3) examinations demonstrate multiple radiolucent lines in the anterior cortex of mid-tibial-diaphysis (dreaded black line sign) resulting from repeated episodes of stress fractures. The arrow indicates the current symptomatic fracture.

Images in longitudinal planes depict the fractures more clearly and confirm partial cortical involvement without extension in the medulla or entire bone width. Undulant bony contours around the fractures due to chronic periosteal reaction is noted, but no acute or subacute periosteal reaction is seen.

## Comments

Stress fractures are usually recognized on X-rays due to focal area of increased density and cortical thickening caused by periosteal reaction around the stress fracture. Closer inspection often reveals the lucent fracture line within the area of focal periosteal reaction.

The “dreaded black line” is a radiological sign for a unique type of stress fracture in the anterior cortex of mid-tibia, seen initially only as a discrete transverse radiolucent (black) line in the anterior cortex of mid-tibia (from where it derives its name). The overall incidence of these fractures is 0.8–7% and they comprise 5–15% of all tibial stress fractures.

Sometimes, these stress fractures may be multiple, as in our case [1]. If it is the first episode, it is imaginable that that this discrete black line can be easily overlooked due to lack of exuberant acute periosteal reaction around them.

Hypovascularity and continuous muscular tension and pull from posterior leg makes the anterior cortex of mid-tibia particularly prone to such stress fractures. For the same reasons, progression to complete fracture, delayed union, and non-union are common complications if overloading is continued [1].

It is important to recognise and treat these fractures early to obviate the “dreaded” complications of this stress fracture. They can be treated conservatively with a non-weight-bearing immobilization for six to eight weeks. A more aggressive surgical treatment with excision and bone grafting or intramedullary rod placement is undertaken early in the course.

Though we could not find the rate of missed fractures or progression of these fractures to complete fractures in literature, a two-case series comparing conservative versus surgical treatment reported non-union in 93% (13/15) and 53% (9/17) with conservative treatment.
